# Testosterone-to-estradiol ratio and platelet thromboxane release in ischemic heart disease: the EVA project

**DOI:** 10.1007/s40618-022-01771-0

**Published:** 2022-03-09

**Authors:** V. Raparelli, C. Nocella, M. Proietti, G. F. Romiti, B. Corica, S. Bartimoccia, L. Stefanini, A. Lenzi, N. Viceconte, G. Tanzilli, V. Cammisotto, L. Pilote, R. Cangemi, S. Basili, R. Carnevale, The EVA Collaborators, Claudio Tiberti, Federica Panimolle, Andrea Isidori, Elisa Giannetta, Mary Anna Venneri, Laura Napoleone, Marta Novo, Silvia Quattrino, Simona Ceccarelli, Eleni Anastasiadou, Francesca Megiorni, Cinzia Marchese, Enrico Mangieri, Gaetano Tanzilli, Nicola Viceconte, Francesco Barillà, Carlo Gaudio, Vincenzo Paravati, Guglielmo Tellan, Evaristo Ettorre, Adriana Servello, Fabio Miraldi, Andrea Moretti, Alessandra Tanzilli, Piergiovanni Mazzonna, Suleyman Al Kindy, Riccardo Iorio, Martina Di Iorio, Gennaro Petriello, Laura Gioffrè, Eleonora Indolfi, Gaetano Pero, Nino Cocco, Loredana Iannetta, Sara Giannuzzi, Emilio Centaro, Sonia Cristina Sergi, Pasquale Pignatelli, Daria Amoroso, Simona Bartimoccia, Giovanni Talerico, Salvatore Minisola, Sergio Morelli, Antonio Fraioli, Silvia Nocchi, Mario Fontana, Filippo Toriello, Eleonora Ruscio, Tommaso Todisco, Nicolò Sperduti, Giuseppe Santangelo, Giacomo Visioli, Marco Vano, Marco Borgi, Ludovica Maria Antonini, Silvia Robuffo, Claudia Tucci, Agostino Rossoni, Valeria Spugnardi, Annarita Vernile, Mariateresa Santoliquido, Verdiana Santori, Giulia Tosti, Fabrizio Recchia, Francesco Morricone, Roberto Scacciavillani, Alice Lipari, Andrea Zito, Floriana Testa, Giulia Ricci, Ilaria Vellucci, Marianna Vincenti, Silvia Pietropaolo, Camilla Scala, Nicolò Rubini, Marta Tomassi, Gloria Rozzi, Floriana Santomenna, Claudio Cantelmi, Giacomo Costanzo, Lucas Rumbolà, Salvatore Giarrizzo, Carlotta Sapia, Biagio Scotti, Danilo Toni, Anne Falcou, Louise Pilote, Amanpreet Kaur, Zhara Azizi, Anna Rita Vestri, Patrizia Ferroni, Clara Crescioli, Cristina Antinozzi, Francesca Serena Pignataro, Tiziana Bellini, Giovanni Zuliani, Angelina Passaro, Brombo Gloria, Andrea Cutini, Eleonora Capatti, Edoardo Dalla Nora, Francesca Di Vece, Andrea D’Amuri, Tommaso Romagnoli, Francesco Luciani, Michele Polastri, Alessandra Violi, Valeria Fortunato, Alessandro Bella, Roberto Manfredini, Alfredo De Giorgi, Fabio Fabbian, Roberto Carnevale, Cristina Nocella, Carlo Catalano, Iacopo Carbone, Nicola Galea, Giuliano Bertazzoni, Marianna Suppa, Antonello Rosa, Gioacchino Galardo, Maria Alessandroni, Alessandro Coppola, Mariangela Palladino, Giulio Illuminati, Fabrizio Consorti, Paola Mariani, Fabrizio Neri, Paolo Salis, Antonio Segatori, Laurent Tellini, Gianluca Costabile

**Affiliations:** 1 Department of Experimental Medicine, Sapienza University of Rome, Rome, Italy; 2 Faculty of Nursing, University of Alberta, Edmonton, AB Canada; 3 Department of Translational Medicine, University of Ferrara, via Luigi Borsari, 46, 44121 Ferrara, Italy; 4 University Center for Studies on Gender Medicine, University of Ferrara, Ferrara, Italy; 5 Department of Clinical Internal, Anesthesiological and Cardiovascular Sciences, Sapienza University of Rome, Rome, Italy; 6 Geriatric Unit, IRCCS Istituti Clinici Scientifici Maugeri, Milan, Italy; 7 Department of Clinical Sciences and Community Health, University of Milan, Milan, Italy; 8 Liverpool Centre for Cardiovascular Science, University of Liverpool and Liverpool Heart and Chest Hospital, Liverpool, UK; 9 Department of Translational and Precision Medicine, Sapienza University of Rome, Rome, Italy; 10 Department of Medico-Surgical Sciences and Biotechnologies, Sapienza University of Rome, Latina, Italy; 11 Department of General Surgery and Surgical Speciality Paride Stefanini, Sapienza University of Rome, Rome, Italy; 12 Centre for Outcomes Research and Evaluation, McGill University Health Centre Research Institute, Montreal, QC Canada; 13 Mediterranea Cardiocentro-Napoli, Naples, Italy

**Keywords:** Testosterone, Estradiol, Ischemic heart disease, Thromboxane, Mortality

## Abstract

**Background:**

Data on the interplay between sexual hormones balance, platelet function and clinical outcomes of adults with ischemic heart disease (IHD) are still lacking.

**Objective:**

To assess the association between the Testosterone (T)-to-Estradiol (E2) Ratio (T/E2) and platelet activation biomarkers in IHD and its predictive value on adverse outcomes.

**Methods:**

The EVA study is a prospective observational study of consecutive hospitalized adults with IHD undergoing coronary angiography and/or percutaneous coronary interventions. Serum T/E2 ratios E2, levels of thromboxane B_2_ (TxB_2_) and nitrates (NO), were measured at admission and major adverse events, including all-cause mortality, were collected during a long-term follow-up.

**Results:**

Among 509 adults with IHD (mean age 67 ± 11 years, 30% females), males were older with a more adverse cluster of cardiovascular risk factors than females. Acute coronary syndrome and non-obstructive coronary artery disease were more prevalent in females versus males. The lower sex-specific T/E2 ratios identified adults with the highest level of serum TxB_2_ and the lowest NO levels. During a median follow-up of 23.7 months, the lower sex-specific T/E2 was associated with higher all-cause mortality (HR 3.49; 95% CI 1.24–9.80; *p* = 0.018). In in vitro, platelets incubated with T/E2 ratios comparable to those measured in vivo in the lowest quartile showed increased platelet activation as indicated by higher levels of aggregation and TxB_2_ production.

**Conclusion:**

Among adults with IHD, higher T/E2 ratio was associated with a lower long-term risk of fatal events. The effect of sex hormones on the platelet thromboxane release may partially explain such finding.

**Supplementary Information:**

The online version contains supplementary material available at 10.1007/s40618-022-01771-0.

## Introduction

Although adverse outcomes from ischemic heart diseases (IHD) have been declining over the last decade, the worldwide burden of IHD remains high in both sexes. Even if females have an age-standardized incidence and prevalence of IHD lower than males [1, 2], the absolute number of females is greater than that of males. Furthermore, sex difference exists in the pathogenesis, progression and response to treatment. Indeed, IHD is not anymore synonymous only with obstructive flow-limiting coronary artery disease (CAD), especially in females that are more commonly affected by non-obstructive disease [3]. Traditionally, biological attributes have been claimed as main drivers of the sex differences across the spectrum of IHD, specifically sex hormones might play a role on the status of the vascular health [4].

Mechanistic understanding of the association between sex steroids and IHD is challenging.

Testosterone (T) and estrogen-related steroid hormones (including 17b estradiol, E2) have been shown to, directly and indirectly affect vascular health [5]. The effects of sex hormones have been analyzed separately with conflicting data in males and females for their protective or harmful effects on cardiovascular health. The disturbance of the physiological balance between E2 and T has been studied to understand its contribution to cardiovascular disease (CVD) progression in a synergistic or co-dependent interplay [6]. For example, in males with severe carotid atherosclerosis, low T/E2 ratio is associated with systemic and plaque inflammation and is a powerful predictor of future cardiovascular events [7]. Interestingly, in females, the reverse regarding T/E2 ratio has been observed, with higher T/E2 ratio being associated with worse CVD outcomes/events in post-menopausal women [8].

In the interplay between sex hormones and vascular health, differences in vasoactive molecules modulated by the T/E2 balance might be relevant. In experimental models, females are at reduced risk of ischemia–reperfusion damage and E2 administered acutely to males can reduce infarct size [9]. The non-genomic effects of sex hormones on vascular cells and platelets recruited at the athero-thrombotic process site are a matter of debate [10, 11]. A sex difference in platelet reactivity has been reported in response to agonists both with and without concomitant antiplatelet therapy [12]. Mechanisms that could account for such differences in platelet biology are mostly unknown. Platelets mainly express estrogen receptor beta (ER-β) on their surfaces [13]. Treating human platelets from healthy men with E2 did not elicit any functional platelet response, but it primed thrombin-induced platelet aggregation through a non-genomic effect [13]. Whether this occurs in platelets from females or in individuals of both sexes with IHD has not been investigated.

In light of this, among adults with IHD we investigated: (i) the relation between sex-specific T/E2 ratio and vasoactive molecules, such as thromboxane B_2_ and NO metabolites; (ii) the existence of an association between sex-specific T/E2 ratio and long-term adverse clinical outcomes; and (iii) whether the association between T/E2 ratio and outcomes can be explained at least in part through sex hormone-dependent effects on platelet function.

## Methods

The data underlying this article will be shared upon reasonable request to the corresponding author.

### Study population

The “Endocrine Vascular disease Approach” (EVA) project (ClinicalTrials.gov identifier NCT02737982), is an observational, prospective study, aimed at exploring sex- and gender-related differences in the interaction between platelet function, sex hormones, and coronary microvascular dysfunction in IHD. The EVA Study design has been previously published [14]. Briefly, EVA enrolled consecutively Italian adults (> 18 years), who were referred to the cardiac catheterization laboratory (cath-lab) undergoing coronary angiography and/or percutaneous coronary intervention for suspected IHD. Based on angiography, IHD patients were classified as follows: (1) ischemia with obstructive CAD, that is, ≥ 50% diameter stenosis; and (2) ischemia with no obstructive CAD (INOCA) < 50% diameter stenosis [15, 16].

During the angiography, blood samples were collected before PCI. Measurements were ascertained while blinded to the sample origin. All samples were assayed in duplicate, and those showing values above the standard curve were retested with appropriate dilutions. According to a previously reported study, the arterial samples are suitable for testing biomarkers of platelet function [17].

The study was conducted in full conformance with the Declaration of Helsinki principles, and it was approved by the local Ethics Committee of Policlinico Umberto I, Sapienza University of Rome, Rome, Italy (reference 3786, 24/09/2015). Written informed consent has been obtained from all patients.

#### Sex-hormone measurements

The concentration of sex hormones was measured in batch by the Laboratory of the Department of Experimental Medicine (Section of Medical Pathophysiology), Sapienza University of Rome in serum samples stored at − 80 °C. Serum E2 and T were measured by chemiluminescent micro-particle immunoassay (CMIA, Architect System) (Abbott Laboratories, IL, USA). The T/E2 ratio was calculated using the following formula: Testosterone/(10*estradiol) as previously reported [7].

#### Vasoactive biomarkers measurements

Serum thromboxane B_2_ (TxB_2_), the stable product of the non-enzymatic hydration of TxA_2_, which itself has a half-life of only 37 s under physiologic conditions, was measured by ELISA (Cusabio, TX, USA) according to the manufacturer instructions and expressed as picograms per milliliter (pg/ml). Intra-assay and inter-assay coefficients of variation were 4.0% and 3.6%, respectively.

Nitric oxide (NO) bioavailability was determined with a colorimetric assay kit (Cell Biolabs, San Diego, California, USA) by measuring the NO metabolites, nitrite and nitrate, in the serum. Intra-assay and inter-assay coefficients of variation were 2.9% and 1.7%, respectively.

#### Platelet preparation and platelet aggregation

To obtain platelet-rich plasma (PRP), citrated [3.8%, 1/10 (v: v)] blood samples from healthy volunteers were centrifuged for 15 min at 180 *g* at room temperature and the supernatant PRP was separated (2 × 10^5^ platelets/μl). Only the top 75% of the PRP was collected, to avoid leukocyte contamination.

PRP samples were incubated with different concentrations of T and E2 to achieve the T/E2 ratio corresponding to the male-specific lowest (11.6, [T] 9.97 nM and [E2] 0.086 nM) and highest (22.2, [T] 18.3 nM and [E2] 0.0824 nM) quartiles and to the female-specific lowest (0.90, [T] 0.52 nM and [E2] 0.0577 nM) and highest (2.71, [T] 1.15 nM and [E2] 0.0424 nM) quartiles. Following incubation, platelets were stimulated with a sub-threshold concentration of collagen (STC, 0.25 μg/ml), defined as the highest concentration that elicited < 20% platelet aggregation. Platelet aggregation was monitored continuously as light transmission increment. Finally, samples were centrifuged for 3 min at 3000 rpm and supernatants and pellets were stored at − 80 °C to measure TxB_2_.

#### ***Platelet TxB***_***2***_*** assay***

Platelet TxB_2_ was evaluated in the platelet supernatant by an ELISA commercial kit (Cusabio, TX, USA), according to manufacturer instructions. The values were expressed as pg/ml. Intra- and inter-assay coefficients of variation for TxB_2_ were < 8% and < 10%, respectively.

#### Follow-up

Participants were followed up by phone and outpatient visits when feasible for at least one year after discharge; after 12 months, phone interviews were conducted periodically each 3 months. In case of events, the adjudication of the adverse clinical outcomes was performed by assessing the medical chart reviews. For the purpose of this analysis, we assessed all-cause mortality, defined as death for any cause during follow-up.

### Statistical analysis

All continuous variables were tested for normality with the Shapiro–Wilk test. Continuous variables with normal distribution were reported as mean ± standard deviation (SD), non-parametric variables as median and interquartile range (IQR). Between-groups comparisons were performed using *T* test for normally distributed variables and using an appropriate non-parametric test for non-normally distributed variables (Mann–Whitney *U* test or Kruskal–Wallis *H* test). Categorical variables were reported as count and percentages. Between-groups comparisons were made using a *χ*^2^ test, or a Fisher’s exact test if any expected cell count was < 5. For categorical variables with more than two possible values, exact *P* values have been estimated according to the Monte Carlo method. Quartiles of T/E2 ratio have been separately computed for males and females to account for the different physiological distribution of sex hormones and then a pooled sex-specific T/E2 ratio variable was used in all the analyses.

Survival curves were formally compared using the log-rank test. Cox proportional hazards analysis was used to calculate the adjusted relative hazards of outcome events. The final multivariable Cox regression model was selected via a purposeful selection of potential confounders. Given the limited number of events, the relationship of interest has been adjusted for age, sex and the type of CAD (obstructive vs. non-obstructive) and the presence of an acute coronary syndrome).

A two-sided *p* value < 0.05 was considered statistically significant. All analyses were performed using SPSS v. 25.0 statistical software (IBM, NY, USA).

## Results

**Fig. 1:**
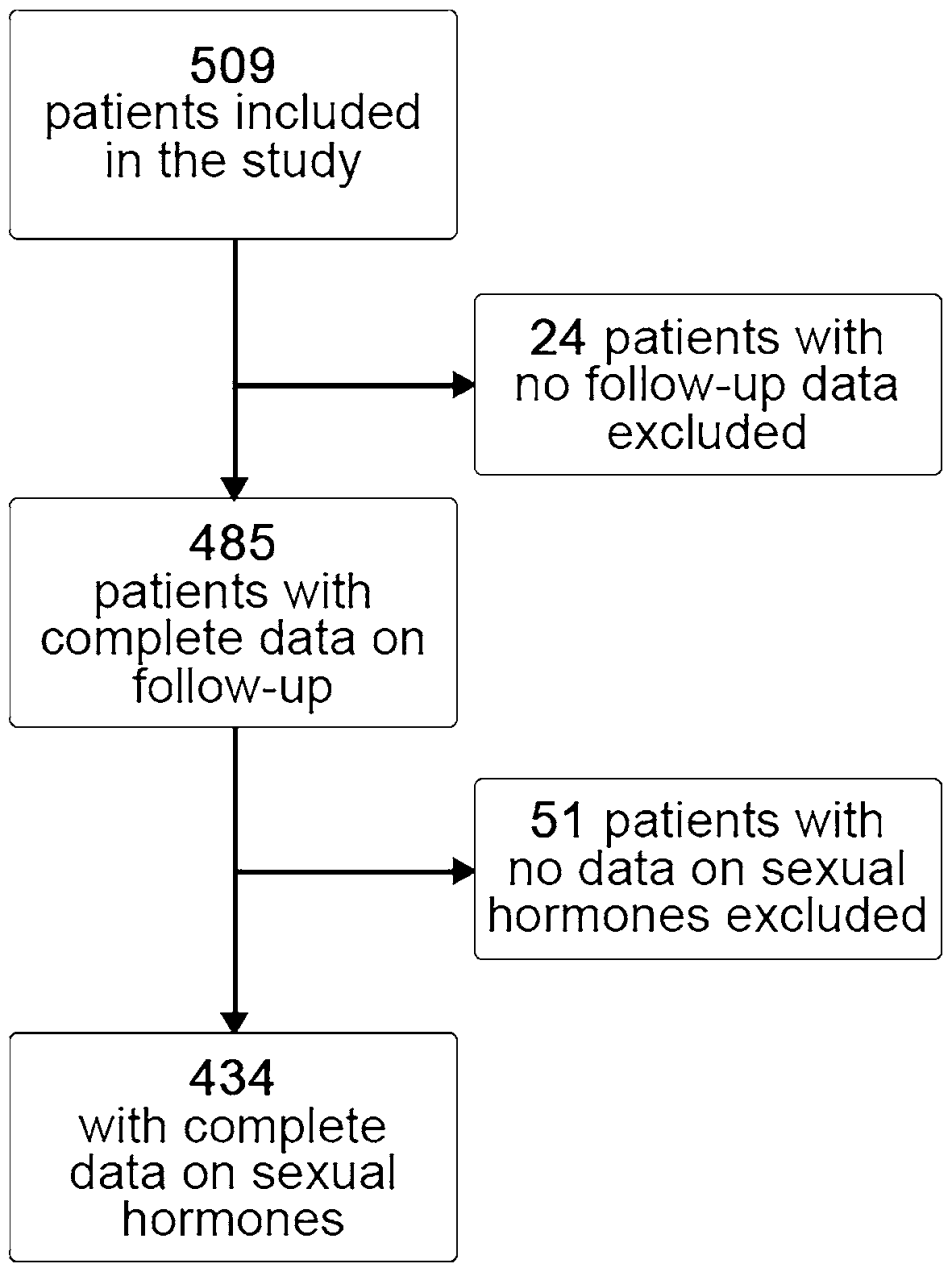
Study population

Among the 509 consecutive adults with IHD recruited in the EVA study (Table S1), 434 (85%) were analyzed as they had both ratio T/E2 measured at baseline and follow-up data (Fig. 1). None of the participants was under hormonal therapies. The baseline clinical characteristics stratified by sex are summarized in Table 1. Males were younger, with a more adverse cluster of cardiovascular risk factors than females. The referral reason for coronary angiography was acute coronary syndrome (ACS) in half of the cases. Females more frequently exhibited non-obstructive coronary artery disease than males.

**Table 1:** Baseline characteristics stratified by sex

Variables	Women (*n* = 133)	Men (*n* = 301)	*p* value
Age (years) mean ± SD	69.8 ± 11.5	65.4 ± 10.7	< 0.001
BMI (kg/m^2^) mean ± SD	26.3 ± 5.0	27.5 ± 4.0	0.009
Family Hx CVD	72 (55.8)	191 (64.7)	0.08
Smoking	25 (19.1)	86 (28.9)	0.031
Hypertension	97 (73.5)	246 (81.7)	0.05
Heart Failure	11 (8.3)	45 (14.9)	0.59
Dyslipidemia	61 (46.2)	162 (54.2)	0.12
Type 2 Diabetes	28 (21.2)	90 (29.9)	0.061
Known IHD	27 (20.4)	127 (42.2)	< 0.001
Prior AMI	18 (13.5)	92 (30.6)	< 0.001
Vascular Disease^#^	27 (20.4)	82 (27.2)	0.13
Prior Stroke/TIA	18 (13.6)	28 (9.3)	0.17
Dementia	1 (0.8)	2 (0.7)	0.91
End Stage Chronic Kidney/Dialysis	0 (0)	6 (1.9)	0.10
COPD	15 (11.4)	32 (10.6)	0.82
Statins at admission	50 (37.9)	148 (49.2)	0.029
Anti-platelets at admission			< 0.001
Single	49 (37.1)	147 (48.8)	
DAPT	8 (6.1)	46 (15.3)	
Acute Coronary Syndrome Yes	78 (58.6)	149 (49.5)	0.07
Type of CAD			< 0.001
Obstructive CAD	83 (62.4)	236 (78.4)	
Non-obstructive CAD	50 (37.6)	65 (21.6)	
Creatinine (mg/dl) mean ± SD	0.85 ± 0.30	1.07 ± 0.54	< 0.001
Platelet Count (× 10^3^) mean ± SD	241.6 ± 71.5	209.8 ± 58.0	< 0.001
Hemoglobin (g/dL) media ± SD	13.1 ± 1.5	14.4 ± 1.6	< 0.001
Estradiol (pg/mL) median [IQR]	10.0 [9.5–17.0]	24.0 [16.0–31.0]	< 0.001
Testosterone (nmol/L) median [IQR]	0.8 [0.5–1.2]	14.1 [9.9–17.6]	< 0.001
T/E2 Ratio median [IQR]	1.8 [0.9–2.71]	15.8 [11.5–22.0]	< 0.001
Thromboxane (pg/ml), median [IQR]*	152.0 [116.0–187.0]	147.3 [96.0–220.6]	0.40
NO (uM) median [IQR]*	21.5 [12.5–30.2]	17.9 [8.1–38.8]	0.39

*AMI* Acute Myocardial Infarction, *BMI* body mass index, *CAD* coronary artery disease, *COPD* chronic obstructive pulmonary disease, *DAPT* dual-antiplatelet therapy, *Hx* history, *PAD* *TIA* transient ischemic attack

*Data available on 428 patients

^#^Peripheral artery disease and/or Carotid Stenosis

The serum levels of TxB_2_ and NO were not different between males and females (Table 1).

The ranges of the sex-specific quartiles of T/E2 ratio were as follows: (1) male group (Q1 < 11.63; Q2 = 11.63–15.93; Q3 = 15.94–22.19; Q4 > 22.19); (2) female group (Q1 < 0.89; Q2 = 0.89–1.76; Q3 = 1.76–2.71; Q4 > 2.71).

**Fig. 2:**
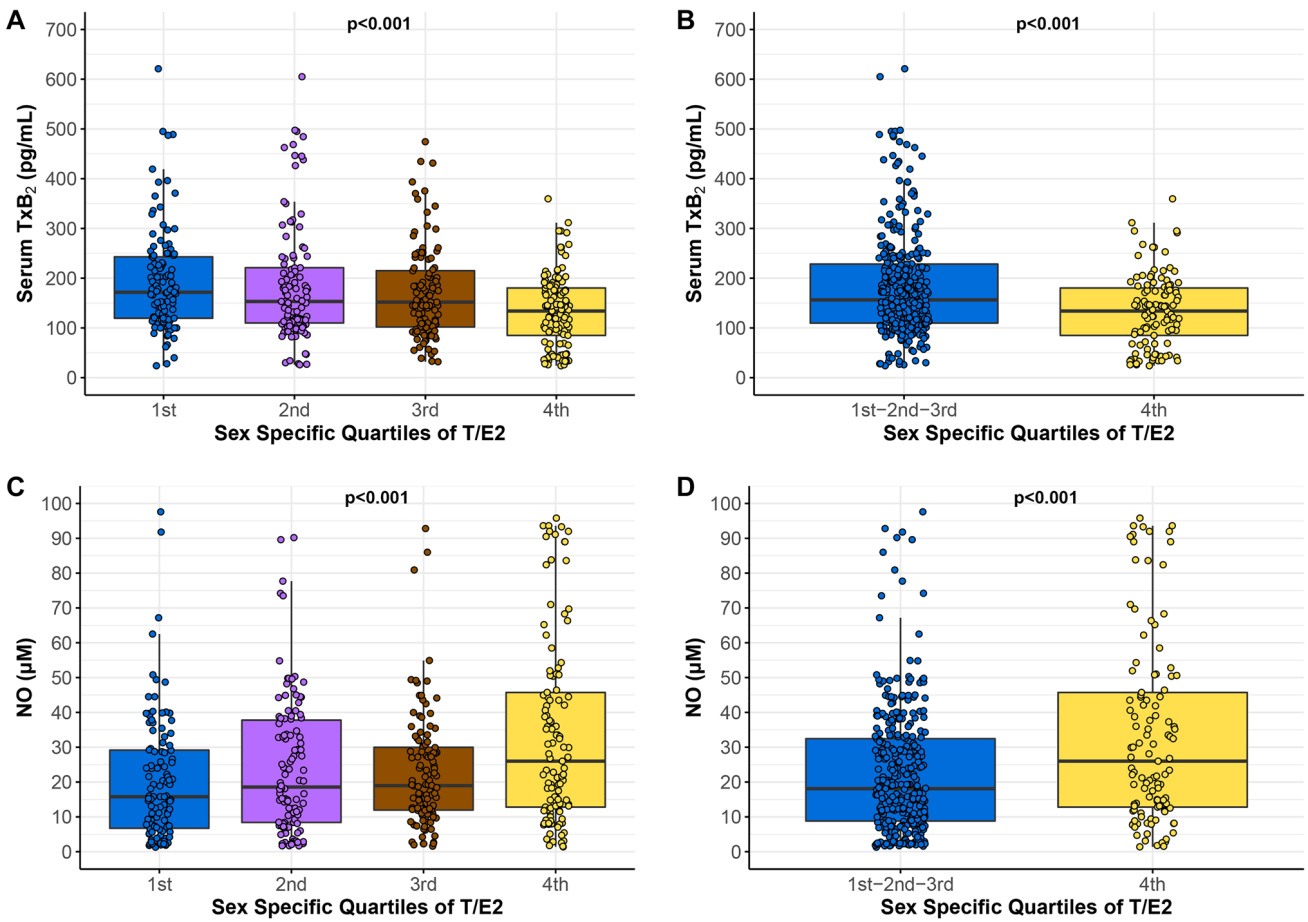
Levels of Vasoactive Molecules by Sex-specific Quartiles of T/E2 Ratio. Serum thromboxane B_2_ (TxB_2_) across quartiles of T/E2 (**A**) and comparing the highest versus the other quartiles of T/E2 ratio (I–II–III vs IV) (**B**); Serum nitric oxide (NO) across quartiles of T/E2 (**C**) comparing the highest versus the other quartiles of T/E2 ratio (I–II–III vs IV) (**D**). TxB_2_ and NO levels were compared with Mann–Whitney *U* (for the comparisons between two groups, I–II–III vs IV) and Kruskal–Wallis *H* (for the comparisons between three or more group)

When considering the entire cohort based on sex-specific quartiles of the T/E2 ratio, we observed a progressive decrease of TxB_2_ and a progressive increase of NO across the increasing T/E2 sex-specific quartiles (Fig. 2A and B). Specifically, participants in Q4 showed lower levels of serum TxB_2_ (134 [78–181] vs. 158 [110–231] pg/ml; *p* < 0.001) and higher levels of NO 26.0 [12.7–46.1] vs. 18.1 [8.7–32.5] µM; *p* < 0.001), compared to the other quartiles (Q1-3) (Fig. 2C and D).

During a median of 23.7 (IQR 11.7–38.8) months of follow-up, 41 patients died (16 cardiac deaths, 3 fatal strokes, 8 cardiovascular deaths, 14 non-vascular deaths).

Patients who died were older (75.5 ± 10.9 vs. 65.9 ± 10.7; *p* < 0.0001), more likely to have obstructive coronary disease (87.8% vs. 72%; *p* = 0.029) and, to have an ACS (65.9% vs. 50.9%; *p* = 0.06), and to be females (43.9% vs. 29.3%; *p* = 0.05).

**Fig. 3:**
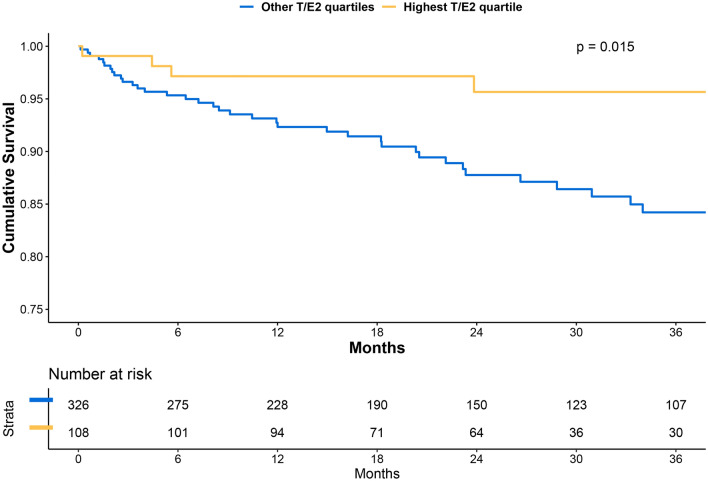
Cumulative Incidence of All-cause Death by Sex-specific T/E2 Quartiles. Log-Rank test was used to compare the two groups

The rate of all-cause death was lower among adults in the sex-adjusted T/E2 highest quartile than those in the other quartiles (Q1-3) (3.7% vs. 11.3%; *p* = 0.015) (Fig. 3).

At univariate Cox regression analyses, the sex-adjusted T/E2, the lower quartiles (Q1-3 vs. Q4 as reference group) (HR 3.333; 95% CI 1.187–9.355; *p* = 0.022), and age (HR 1.103; 95% CI 1.064–1.143; *p* < 0.001) were significantly associated with mortality; female sex (HR 1.668; 95% CI 0.929–3.098; *p* = 0.086), ACS presentation (HR 1.788; 95% CI 0.943–3.390), and obstructive CAD (HR 2.514; 0.986–6.408; *p* = 0.053) showed a tendency to be associated with mortality.

A multivariable Cox regression analysis showed that the lower quartiles of sex-adjusted T/E2 (HR 3.487; 95% CI 1.241–9.798; *p* = 0.018) and age (HR 1.100; 95% CI 1.062–1.138; *p* < 0.001) were independently associated with mortality after adjusting for sex, ACS presentation and obstructive type of CAD).

### In vitro study

**Fig. 4:**
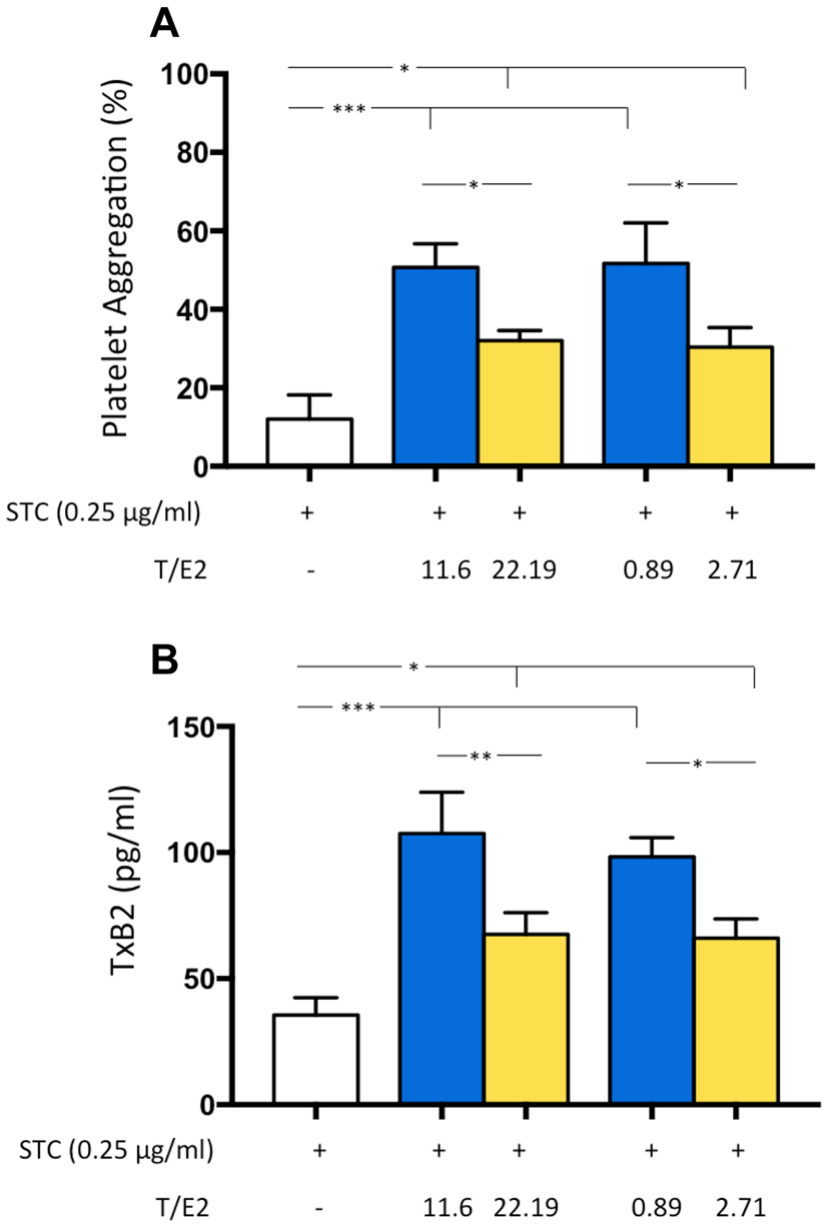
Sex-specific Quartiles of T/E2 Ratio and Platelet Activation. Platelet aggregation (**A**) and TxB2 biosynthesis (**B**) were evaluated in platelets from healthy subjects incubated with different T/E2 ratio, 11.63 or 22.19 corresponding to the lowest and highest quartiles of the T/E2 ratio for male and 0.897 or 2.71 corresponding to the lowest and highest quartiles of the T/E2 ratio for female, before stimulation with a subthreshold concentration of collagen (STC, 0.25 μg/ml). Data shown are means ± SD and were compared with Mann–Whitney *U* (for the comparisons between two groups) and Kruskal–Wallis *H* (for the comparisons between three or more group). *p < 0.05; **p < 0.01; ***p < 0.001

To confirm the interplay of sex-adjusted T/E2 ratio and platelet function, platelets from healthy volunteers (3 males, mean age 60.7 ± 6.0 and 3 females, mean age 63.3 ± 4.2) were incubated with sex-adjusted quartiles of the T/E2 ratio and stimulated with STC of collagen. Priming platelets with T/E2 ratio resulted in a strong potentiation of platelet aggregation and TxB_2_ biosynthesis induced by STC of collagen, as shown in Fig. 4. However, the sex-adjusted T/E2 Q4 induced less pronounced priming of platelet activation.

## Discussion

In an Italian cohort of consecutive hospitalized patients with IHD, the sex-specific lower T/E2 ratios were associated with an increased risk of adverse fatal event during a long-term follow-up. The association with fatal events persisted after adjustment for age, clinical presentation, and type of CAD. The assessment of in vivo vasoactive molecules and the in vitro experiments demonstrated that the lower sex-specific T/E2 ratios induced an increase in platelet thromboxane release and a decrease in NO bioavailability that may partially explain why endogenous sex hormones favors the occurrence of fatal adverse events.

Despite advancements in diagnostics and therapeutics, IHD has a high burden globally both in males and females, CVD being the leading cause of mortality worldwide [18]. The role of sex hormones and their implications in vascular health is a matter of debate in the scientific community [19]. Despite biologically plausible pathological pathways linking endogenous sex hormones and clinical adverse events, prospective studies have yielded conflicting results [8, 20]. Of note, the same category of sex hormones exerts a different effect depending on the individual’s sex. For example, in males, several studies have demonstrated the increased endogenous T levels, as well as elevated luteinizing hormone and sex hormone-binding globulin, were associated with a decrease in CV events [21, 22]. Conversely, among female individuals, some studies reported a protective effect of elevated E2 levels in terms of IHD development and progression while an increased risk with elevated FSH [23]. The number of challenges faced in understanding the interrelations between sex hormones, biological sex, and vascular health has fostered a research approach based on the use of ratio between T and E2 rather than the absolute concentration of individual hormones. In fact, a balanced T/E2 ratio is needed for contrasting early-stage atherosclerosis [24]. Accordingly, we used such approach in a cohort of hospitalized female and male individuals with IHD to assess the sex-specific effect of T/E2 ratios on vasoactive biomarkers that reflect the propensity to develop myocardial ischemia. The lower sex-specific T/E2 ratios among adults with ischemia resulted in higher TxB_2_ and lower NO levels, suggesting that the contribution of sex hormones is mediated, at least in part, by their effect on vasoactive molecules.

However, the vasoactive theory is not the only one that could explain the increased platelet reactivity. Platelets from females are more reactive in response to various agonists than those of male counterparts [12, 25]. Sex hormones may partially be responsible for such sex difference in platelet function. It has been suggested that sex hormones exert either a non-genomic effect directly on platelets or a genomic effect on megakaryocytes. Administration of testosterone to healthy men was shown to increase the thromboxane A2 receptor expression and was associated with an increased platelet activation [26]. Furthermore, the impact of reproductive age (i.e., pre- versus post-menopause), as well as the effect of hormone replacement therapy in post-menopause on female platelets has been explored both in experimental models and humans although with discordant results, likely related with the different approaches used for assessing the platelet function (e.g., flow cytometry or surrogate markers such as mean platelet volume). In fact, while initially the platelet activation assessed in female subjects through flow cytometry resulted in conflicting data [27, 28], later on evidence supported that proteins [29], mRNAs and miRNAs [30] are differentially expressed by sex in platelets and changes in platelet reactivity in relation to the hormonal status. Of note, administration of estradiol to mice has been shown to change the expression level of the collagen receptor GPVI [31] and of the cytoskeletal protein beta-tubulin [32]. The advantage of using sex-adjusted T/E2 ratios is to account for the well-known differences in sex hormone system along the lifespan of individuals, regardless of the reproductive age.

The mechanisms responsible for the detrimental effect of the sex-specific lower T/E2 ratios on vascular health and clinical outcomes are not clearly elucidated. Recently, clinical studies have also explored the contribution of testosterone in cardiovascular disease [33] sometimes with contradictory results. The MrOS prospective study of 552 elderly male patients found no relationship between sex hormones levels (i.e., testosterone and T/E2 ratio quartiles) and the risk of cardiovascular events [34]. Conversely, our study includes younger patients of both sexes, and we found that lower T/E2 quartiles were associated with increased risk of all-cause death. Taken together, these results may underlie how the effect of sex hormones can be different across age strata, perhaps with increased importance in younger cohorts. However, further confirmation is needed to support this hypothesis. Indeed, in the MESA (Multi-Ethnic Study of Atherosclerosis) study among racially/ethnically diverse 2834 post-menopausal women with long-term follow-up, a higher T/E2 ratio was associated with an elevated risk for incident CVD [8]. In contrast, in men, the opposite pattern is seen with low testosterone being associated with endothelial dysfunction and CHD. Prior work has shown that in males with atherosclerotic disease, the impairment of vascular health has been mainly related to inflammatory milieu and to the endocrine function. In fact, among 611 male carotid endarterectomy patients included in the Athero-Express Biobank Study, low T/E2 ratio was associated with increased systemic inflammation and an increased risk of future CV events [7]. Notably, the BMI resulted as an important effect modifier of T/E2 ratio effect on future events, suggesting that the aromatase activity that produces E2 via conversion of T in the visceral/abdominal adipose tissue can be linked to a pro-inflammatory and more vulnerable pattern of the atherosclerotic disease. Beyond the metabolic pattern, we hypothesized that activated platelets, which are major pathogenetic players in myocardial ischemia and express sex hormone receptors on their surface, release potent molecules favoring vasoconstriction, explaining at least in part the effect of sex hormone balance on vascular status. More specifically, platelets have been reported to contribute to the coronary microvascular dysfunction by several mechanisms that include forming distal micro-emboli, adhering to re-perfused endothelium, releasing vasoconstrictor or toxic molecules and inflammatory mediators that further enhance the activation of the endothelial monolayer and the recruitment of circulating neutrophils [35–37]. Hyperactive platelets are therefore implicated in the pathogenesis of ischemia. Of note, the platelet function has been reported to be different between males and females [38, 39]. Of note, according to the available literature, platelets from men and women express comparable levels of AR and ERbeta and of COX1 [40]. However, testosterone regulates AR expression in megakaryocytes in a non-linear manner (i.e., low testosterone upregulates AR, while high testosterone downregulates AR) and this may account at least in part for the different effects of sex hormones. Moreover, the non-genomic signaling pathways activated by these receptors are still poorly understood. Thus, we cannot exclude that sex differences in the expression of signaling proteins downstream of these receptors may contribute to the different effects.

Among molecules released during platelet activation, TxB_2_ production represents an amplification mechanism of platelet aggregation. As we observed in vivo that the lower sex-adjusted T/E2 ratios among adults with ischemia resulted in higher TxB_2_, we performed in vitro experiments to better define the role of sex hormones in platelet activation. We found that the incubation of human platelets with sex-specific quartiles of T/E2 results in a strong potentiation of platelet aggregation and TxB_2_ biosynthesis confirming the role of sex-specific T/E2 in modulating platelet function. However, we observed that the highest sex-adjusted T/E2 quartile induced less pronounced priming of platelet activation, suggesting that the strategy to optimize the balance between sex hormone may play a role in the regulation of platelet function.

### Strengths and limitations

The present study has several strengths worth mentioning. Through a translational approach, we provided sex-specific evidence that supported a platelet-mediated mechanism on how endogenous hormones affect vascular health and consequently clinical outcome. Fostering the understanding of sex disparities in adverse outcomes among individuals with IHD is a remarkable priority to reduce the global burden of CVD and achieve equity in cardiovascular health [3, 18].

This study should be interpreted in the context of several limitations. As any observational cohort study, some confounders might not have been assessed and could influence our final multivariate model, as could any missing data. The findings refer to a selected cohort of Italian individuals at very high cardiovascular risk, hospitalized for an ischemic event, at a single center, limiting the generalizability of our results. A larger participation of female in our study and a higher rate of adverse events would have increase the clinical relevance of our findings. Also, due to sample size limitation, we were unpowered to explore whether the relationship between T/E2 ratio and platelet activations may differ in patients with obstructive vs. non-obstructive CAD; further studies are required to evaluate whether significant related differences may exist. Moreover, due to the paucity of blood samples for some participants, we could not assess the sex hormones balance in the original cohort of 509 participants. Finally, local metabolism of testosterone to estrogen by aromatase in cardiac tissue [41] adds complexity in the understanding hormonal modulation of cardiac and coronary vascular function. Unfortunately, serum concentrations of the hormones may not also reflect the contribution of hormones tissue metabolism.

Finally, vasoactive molecules other than platelet TxB_2_ biosynthesis and NO should be investigated along with the contribution of other cellular lines, such as endothelial cells, to better define the effect of sex hormones balance on platelet function.

## Conclusion

Among individuals with IHD, the sex-specific higher T/E2 ratio was associated with a lower long-term risk of fatal events among adults with IHD. The effect of the T/E2 ratio on the platelet TxB_2_ release may partially explain such finding and foster the development of sex-specific tailored strategy to counteract hormone-related platelet activity. Therefore, further larger prospective clinical study should be designed to assess whether measuring endogenous sex hormones, regardless of age, can contribute to a better sex-specific stratification and management of adverse clinical outcomes in individuals with IHD.

## Supplementary Information

Below is the link to the electronic supplementary material.

Supplementary file1 (DOCX 17 KB)
