# Supplementary material for: Testosterone-to-estradiol ratio and platelet thromboxane release in ischemic heart disease: the EVA project
Source: J Endocrinol Invest. 2022 Mar 9;45(7):1367–77. doi: 10.1007/s40618-022-01771-0 (PMC9184432; doi:10.1007/s40618-022-01771-0)
Supplement: Supplementary file 1 — Supplementary file1 (DOCX 17 KB) [file 40618_2022_1771_MOESM1_ESM.docx]

**SUPPLEMENTAL MATERIAL**

**Table S1. Baseline Characteristics of the Overall EVA cohort Stratified by Sex**

| **Variables** | **Women**  **(n=151)** | **Men**  **(n=358)** | **P-value** |
| --- | --- | --- | --- |
| **Age (years)** **mean ± SD** | 69.8 ± 11 | 65.4 ± 11 | <.001 |
| **BMI (kg/m^2^)** **mean ± SD** | 26.1 ± 5.0 | 27.4 ± 4.0 | 0.014 |
| **Family Hx CVD** | 80 (54.8) | 215 (62.7) | 0.10 |
| **Smoking** | 29 (19.6) | 104 (29.9) | 0.018 |
| **Hypertension** | 110 (73.3) | 293 (81.8) | 0.031 |
| **Heart Failure** | 14 (9.3) | 48 (13.5) | 0.19 |
| **Dyslipidemia** | 66 (44.0) | 193 (54.2) | 0.036 |
| **Type 2 Diabetes** | 32 (21.3) | 105 (29.3) | 0.064 |
| **Known IHD** | 32 (21.3) | 144 (40.2) | <.001 |
| **Prior AMI** | 19 (12.6) | 107 (29.9) | <.001 |
| **Vascular Disease^#^** | 29 (19.3) | 93 (25.9) | 0.11 |
| **Prior Stroke/TIA** | 20 (13.3) | 36 (10.1) | 0.28 |
| **Dementia** | 2 (1.3) | 2 (0.6) | 0.37 |
| **End Stage Chronic Kidney/Dialysis** | 0 (0) | 9 (2.5) | 0.05 |
| **COPD** | 16 (10.7) | 34 (9.5) | 0.69 |
| **Statins at admission** | 53 (35.3) | 171 (47.8) | 0.010 |
| **Antiplatelets at admission** |  |  | <.001 |
| **- Single** | 54 (36) | 173 (48.3) |  |
| **- DAPT** | 10 (6.6) | 53 (14.8) |  |
| **Acute Coronary Syndrome Yes** | 88 (34.11) | 170 (65.9) | 0.026 |
| Type of CAD |  |  | 0.001 |
| - Obstructive CAD | 94 (62.3) | 274 (76.5) |  |
| - Non-obstructive CAD | 57 (37.7) | 84 (23.5) |  |
| **Creatinine (mg/dl) mean ± SD** | 0.85±0.3 | 1.07±0.5 | <.001 |
| **Platelet Count (x10^3) mean ± SD** | 242.2±58.0 | 210.1±69.8 | <.001 |
| **Hemoglobin (g/dL) median [IQR]** | 13.1 ± 1.6 | 14.4 ± 1.6 | <.001 |
| **Estradiol (pg/mL) median [IQR]** | 10 [9.5-18] | 24 [16-31] | <.001 |
| **Testosterone (nmol/L) median [IQR]** | 0.85 [0.52-1.15] | 14.3 [9.97-18.3] | <.001 |
| **T/E2 Ratio median [IQR]** | 1.76 [0.90-2.71] | 15.93 [11.6-22.2] | <.001 |
| **Thromboxane (pg/ml), median [IQR]*** | 149.0 [117.3-190.0] | 147.3 [96.5-220.1] | 0.39 |
| **NO (uM) median [IQR]***** | 20.9 [12.5-30.0] | 18.2 [8.4-38.5] | 0.49 |

Legend: AMI, Acute Myocardial Infarction; BMI, body mass index; CAD, coronary artery disease; COPD, chronic obstructive pulmonary disease; DAPT, dual antiplatelet therapy; Hx, history; PAD, TIA, transient ischemic attack;

*****Data available on 469 patients (144 women, 325 men)

^#^Peripheral artery disease and/or Carotid Stenosis.
